# Validation of self-administered nasal swabs and postage for the isolation of *Staphylococcus aureus*

**DOI:** 10.1099/jmm.0.000381

**Published:** 2016-12-16

**Authors:** Ewan M. Harrison, Nicholas S. Gleadall, Xiaoliang Ba, John Danesh, Sharon J. Peacock, Mark Holmes

**Affiliations:** ^1^​ Department of Veterinary Medicine, University of Cambridge, Cambridge, UK; ^2^​ Department of Public Health and Primary Care, University of Cambridge, Cambridge, UK; ^3^​ Wellcome Trust Sanger Institute, Hinxton, UK; ^4^​ Department of Medicine, University of Cambridge, Cambridge, UK

**Keywords:** Staphylococcus aureus, swabbing, colonisation, carriage, self-swabbing

## Abstract

*Staphylococcus aureus* carriers are at higher risk of *S. aureus* infection and are a reservoir for transmission to others. Detection of nasal *S. aureus* carriage is important for both targeted decolonization and epidemiological studies. Self-administered nasal swabbing has been reported previously, but the effects of posting swabs prior to culture on *S. aureus* yield have not been investigated. A longitudinal cohort study was performed in which healthy volunteers were recruited, trained in the swabbing procedure and asked to take weekly nasal swabs for 6 weeks (median: 3 weeks, range 1–6 weeks). Two swabs were taken at each sampling episode and randomly assigned for immediate processing on arrival to the laboratory (Swab A) or second class postage prior to processing (Swab B). *S. aureus* was isolated using standard methods. A total of 95 participants were recruited, who took 944 swabs (472 pairs) over a median of 5 weeks. Of these, 459 swabs were positive for *S. aureus*. We found no significant difference (*P*=0.25) between 472 pairs of nasal self-swabs processed immediately or following standard postage from 95 study participants (51.4 % vs. 48.6 %, respectively). We also provide further evidence that persistent carriers can be detected by two weekly swabs with high degrees of sensitivity [92.3 % (95 % CI 74.8–98.8 %)] and specificity [95.6 % (95 % CI 84.8–99.3 %)] compared with a gold standard of five weekly swabs. Self-swabbing and postage of nasal swabs prior to processing has no effect on yield of *S. aureus*, and could facilitate large community-based carriage studies.

## Introduction

Around 30 % of humans carry *Staphylococcus aureus*, with the anterior nares being the most common site of colonization (Williams, 1963; Peacock *et al.*, 2001). *S. aureus* carriage has been categorized over many years as persistent (approximately 20 %), intermittent (approximately 30 %) or absent (approximately 50 %), although recent evidence categorizes carriage as a binary characteristic (persistent carriers and the remainder) (Williams, 1963; van Belkum *et al.*, 2009). Persistent carriers have higher bacterial loads than intermittent carriers (Nouwen *et al.*, 2004), are at higher risk of infection (van Belkum *et al.*, 2009) and represent a reservoir for transmission and outbreaks (Harris *et al.*, 2013). Screening and decolonization of methicillin-resistant *S. aureus* (MRSA) carriers before and during hospital admission are performed to reduce the rate of nosocomial MRSA infection (Huang *et al.*, 2013). Detection of carriage is also important for population-level studies of *S. aureus* carriage and molecular epidemiology (Miller *et al.*, 2014; Votintseva *et al.*, 2014). Carriage is detected by taking a swab of the anterior nares or by multi-site swabbing (axilla, groin and nasal swabs). Medical personnel usually perform swabbing, but an increasing number of studies are making use of self-administered nasal swabs (self-swabbing) (Akmatov & Pessler, 2011; van Cleef *et al.*, 2012; Gamblin *et al.*, 2013; Akmatov *et al.*, 2014; Coughtrie *et al.*, 2014; Votintseva *et al.*, 2014). Self-swabbing combined with postage to the processing laboratory would facilitate large-scale epidemiological studies (van Cleef *et al.*, 2010; Akmatov & Pessler, 2011), but, to our knowledge, no studies have validated the effect of postage on the sensitivity of *S. aureus* isolation. Here, we describe a study that addresses this question, and show that self-administered swabbing followed by postage has no significant effect on *S. aureus* yield.

## Methods

### Ethics.

The study was approved by the Ethical Review Committee of the Department of Veterinary Medicine, University of Cambridge (CR116).

### Study cohort and protocol.

Participants were recruited from staff and students in the Department of Veterinary Medicine, University of Cambridge. After informed consent, each participant was given a pack containing 12 Transtube swabs with liquid Amies (MWE), preprepared unique study identifier stickers, a short questionnaire requesting information on gender and age range, and written instructions for self-swabbing together with a URL link (https://www.youtube.com/watch?v=hOEoakxVnfw) to a YouTube video demonstrating the nasal self-administered swabbing technique. Participants were asked to report any antibiotic usage during the study. Participants were asked to take two consecutive self-administered nasal swabs of both nostrils, once weekly for 6 weeks, and to attach stickers identifying which swab was taken first (Swab 1) and second (Swab 2). Participants then deposited swabs on the day of sampling in a central collection point (at room temperature). Swabs were then collected throughout the day and taken to the processing laboratory within the department for processing. Swabs were processed on the same day that they were collected from the collection point. Swabs from each participant were then randomly assigned for either immediate processing, or processing after postage via second class post back to the department.

### Swab processing and *S. aureus* culture.

Swabs in their transport tubes were briefly vortexed and the n inoculated into 4 ml nutrient broth with 7.5 % salt (Oxoid), which was incubated statically in air for 18 h at 37 °C. A 100 µl sample was then plated onto Brilliance Staph 24 agar (Oxoid) and incubated in air for 24 h at 37 °C. Negative plates were incubated for a further 24 h. Blue colonies were streaked to purity on Brilliance Staph 24 agar and presumptive *S. aureus* identity confirmed by *femB* colony PCR (Paterson *et al.*, 2014).

### Statistical analysis.

The statistical analysis was performed using R (R Foundation for Statistical Computing, Vienna, Austria: http://www.R-project.org/). Fisher’s exact test was used to compare two proportions. Binomial logistic regression was used to fit the data to a generalized linear mixed-effects model to examine the effect of subject, postage (posted vs processed immediately), time (week) and swabbing sequence (Swab 1 or 2) on the likelihood of a positive isolation of *S. aureus* from a swab. In a mixed model, individual subjects were treated as a random effects and the remaining parameters as fixed effects.

## Results

### Validation of self-swabbing and postage

A total of 95 participants were recruited, who took 944 swabs (472 pairs) during a median of three consecutive weeks (range 1–6 weeks) (Table 1). Ninety participants provided information on gender and age; 35 (39 %) were males, and the group had an age distribution as follows: 18–25 (*n*=38); 26–35 (*n*=21); 36–45 (*n*=13); 46–55 (*n*=15); 56–65 (*n*=3). The median time of transit for swabs that were posted was 2 days (range 2–4 days). A total of 459 swabs were positive for *S. aureus*, of which 236 were processed immediately (Swab A) and 223 were posted prior to processing (Swab B). The point prevalence for *S. aureus* carriage of the 95 participants in the first week was 54.7 %. The yield of *S. aureus* from Swab A and Swab B was 51.4 % [95 % confidence interval (CI) 46.8–55.9 %] and 48.6 % (95 % CI 44.1–53.2 %) (Tables 1and 2), respectively. There were 371 (78.6 %) concordant pairs and 101 (21.4 %) swab pairs with discordant results (one swab negative and the other positive). Of the discordant pairs, 44 (43.6 %, 95 % CI 34.3–53.3 %) were negative for Swab A and positive for Swab B, and 57 (56.4 %, 95 % CI 46.7–65.7 %) were positive for Swab A and negative for Swab B. Positivity of the 237 first and 222 second swabs taken at the same sampling episode (Swabs 1 and 2) was 51.6 % (95 % CI 44.1–53.2 %) and 48.4 % (95 % CI 43.9–53.0 %), respectively (Tables 1 and 2). Statistical analysis using a binomial logistic regression failed to identify any predictor variable other than the individual subjects (*P*=6.3×10^−10^) as being a statistically significant factor associated with the likelihood of a positive isolation of *S. aureus* from a swab. In a generalized linear mixed-effects model with subjects treated as a random effect, the *P* values for fixed effects were: postage *P*=0.25, swab sequence *P*=0.18 and week *P*=0.06, thus excluding any statistically significant effect of postage on the viability of *S. aureus* transported on swabs.

**Table 1.** Number of positive swabs immediately processed (Swab A) and posted and then processed (Swab B) over the 5 weeks of the study Swab 1 and Swab 2 are the first and second, respectively, of two sequentially taken swabs. Percentages are shown in parentheses.

Week	Swab A		Swab B		Total for week
	Swab 1	Swab 2	Swab 1	Swab 2	
1	30 (28.3)	22 (20.8)	24 (22.6)	30 (28.3)	106
2	24 (28.6)	21 (25.0)	17 (20.2)	22 (26.2)	84
3	22 (28.2)	18 (23.7)	18 (23.7)	18 (23.7)	76
4	20 (22.5)	17 (19.1)	13 (14.6)	21 (23.6)	71
5	12 (18.2)	19 (28.8)	25 (37.9)	10 (15.2)	66
6	19 (33.9)	12 (21.4)	13 (23.2)	12 (21.4)	56
Totals	127 (27.6)	109 (23.7)	110 (23.9)	113 (24.6)	459

**Table 2.** Number of swabs immediately processed (Swab A) and posted and then processed (Swab B) over the 5 weeks of the study for all swabs and for persistent carriers only Swab 1 and Swab 2 are the first and second, respectively, of two sequentially taken swabs. Percentages are shown in parentheses.

	All swabs (*n*=459)	Persistent carriers
Immediately processed	236 (51.4 %)	147 (51.9 %)
Posted and processed	223 (48.6 %)	136 (48.1 %)
Swab 1	237 (51.6 %)	147 (51.9 %)
Swab 2	222 (48.4 %)	136 (48.1 %)

### Detection of persistent *S. aureus* carriers

Based on the findings of previous studies reporting that persistent carriage can be defined on the basis of five swabs, we evaluated 71 participants who completed 5 weeks of swabbing (Nouwen *et al.*, 2004; van Belkum *et al.*, 2009). Participants were assigned as persistent carriers (four or more positive), intermediate carriers (one to three positive) or non-carriers (no positives) based on the results of Swab A. There were 26 (36.6 %, 95 % CI 23.7–45.2 %) persistent carriers, 32 (45.1 %, 95 % CI 34.1–56.6 %) intermediate carriers and 13 (18.3 %, 95 % CI 11.0–28.8 %) non-carriers. We then analysed the data from only the persistent carriers to see how postage affected their classification. Persistent carriers had a total of 283 positive swabs, of which 147 (51.9 %, 95 % CI 46.1–57.7 %) were processed immediately and 136 (48.1 %, 95 % CI 42.3–53.9 %) were processed after posting (Table 2). This result closely mirrors the results for the entire cohort, and again there was no statistically significant difference comparing the rates of positivity for posted and immediately processed swabs from persistent carriers with rates from non-persistent carriers (Fisher’s exact *P*=0.85).

The use of two nasal swabs taken a week apart by medical staff has been reported to identify persistent carriers with high specificity and sensitivity (Nouwen *et al.*, 2004; Verhoeven *et al.*, 2012). We investigated how accurately our own data for Swab A would identify persistent carriers using the first two or three swabs versus five weekly swabs as the gold standard. This showed that using two weekly swabs had a sensitivity of 92.3 % (95 % CI 74.8–98.8 %) and a specificity of 95.6 % (95 % CI 84.80–99.3 %), which is consistent with previous reports (Nouwen *et al.*, 2004; Verhoeven *et al.*, 2012). Positive and negative predictive values were 92.3 % (95 % CI: 74.8–98.8 %) and 95.6 % (95 % CI: 84.8–99.3 %), respectively. Three weekly swabs gave a sensitivity of 92.3 % (95 % CI: 74.8–98.8 %) and an improved specificity of 100 % (95 % CI: 92.0–100.0 %). Positive and negative predictive values were 100.0 % (95 % CI: 85.6–100.0 %) and 95.7 % (95 % CI: 85.4–99.3 %), respectively.

## Discussion

We sought to determine whether postage affected the isolation of *S. aureus.* Our results from randomly assigned swabs immediately processed and those posted self-administered swabs show almost equal proportions of positivity, demonstrating that postage has no statistically significant effect on *S. aureus* viability. We also showed that two weekly self-administered nasal swabs were sufficient to detect persistent carriers with a high degree of sensitivity and specificity, as previously reported for studies in which medical staff took the swabs (Nouwen *et al.*, 2004; Verhoeven *et al.*, 2012). Three consecutive weekly swabs offered a small increase in specificity (from 95.6 % to 100.0 %).

Our study had several limitations. Staff and students of a university veterinary medicine department are not a representative demographic of the general population, and their scientific training might affect their approach to self-sampling. Indeed, the first swabs taken by the 95 participants gave point prevalence for *S. aureus* carriage of 54.7 %. This is considerably higher than the point prevalence of 32 and 32.5 %, from two recent studies that sampled geographically distinct adult populations in England (recruited via general practitioners) (Gamblin *et al.*, 2013; Miller *et al.*, 2014). The reasons for the higher carriage rate are not clear, although our cohort had a number of risk factors previously associated with higher carriage, including a high proportion of young adults, and contact with healthcare systems (the veterinary hospital) and livestock (Wertheim *et al.*, 2005; Mollema *et al.*, 2010; Verkade *et al.*, 2013). Furthermore, eight participants reported antibiotic use during the study (all were intermediate carriers), which could have negatively affected *S. aureus* carriage in these individuals. However, a higher prevalence rate in the sampled population is unlikely to have affected an evaluation of the effect of postage. It was also not possible to determine whether know if participants had followed instructions and deposited the swabs on the same day.

Although it would be expected that the swabs that were immediately processed might represent the ‘gold standard’ (i.e. the most accurate result), there were similar numbers of discordant swab pairs for those that became positive after posting (44 % and 43.6 % 95 % CI 34.3–53.3 %) swabs that were negative for the unposted swab but positive when posted (57 % and 56.4 % 95 % CI 46.7–65.7 %). The multivariable analysis gave a *P* value of 0.25 for this effect. The order in which the swabs were taken might also have affected positivity. Of the 236 immediately processed swabs testing positive, 127 (53.8 %, 95 % CI 47.4–60.1 %) were the first swab taken and 109 (46.2 %, 95 % CI 40.0–52.6 %) were second swabs (not statistically significant, *P*=0.17). Therefore it is unlikely that the order the in which swabs were taken had any significant effect on the results presented here.

Taken together, our results provide evidence that self-administered swabbing followed by return by post is a robust and reliable strategy for the detection of *S. aureus* carriers. This could be of particular utility for large, community-based epidemiological studies.
